# Maize stalk rot caused by *Fusarium graminearum* alters soil microbial composition and is directly inhibited by *Bacillus siamensis* isolated from rhizosphere soil

**DOI:** 10.3389/fmicb.2022.986401

**Published:** 2022-10-20

**Authors:** Kang Zhang, Liming Wang, Helong Si, Hao Guo, Jianhu Liu, Jiao Jia, Qianfu Su, Yanbo Wang, Jinping Zang, Jihong Xing, Jingao Dong

**Affiliations:** ^1^State Key Laboratory of North China Crop Improvement and Regulation, Hebei Agricultural University, Baoding, China; ^2^Hebei Key Laboratory of Plant Physiology and Molecular Pathology, Hebei Agricultural University, Baoding, China; ^3^Institute of Plant Protection, Jilin Academy of Agricultural Sciences, Gongzhuling, China; ^4^Maize Research Institute, Liaoning Academy of Agricultural Sciences, Shenyang, China

**Keywords:** *Fusarium graminearum*, rhizosphere soil, microbial diversity, *Bacillus siamensis*, biocontrol

## Abstract

Maize stalk rot caused by *Fusarium graminearum* can reduce the yield of maize and efficiency of mechanized harvesting. Besides, deoxynivalenol and zearalenone toxins produced by *F. graminearum* can also affect domestic animals and human health. As chemical fungicides are expensive and exert negative effects on the environment, the use of biological control agents has become attractive in recent years. In the present study, we collected rhizosphere soil with severe stalk rot disease (ZDD), the rhizosphere soil with disease-free near by the ZDD (ZDH), and measured rhizosphere microbial diversity and microbial taxonomic composition by amplicon sequencing targeting either bacteria or fungi. The results showed that *Fusarium* stalk rot caused by the *Fusarium* species among which *F. graminearum* is frequent and can reduce the abundance and alpha diversity of rhizosphere microbial community, and shift the beta diversity of microorganisms. Furthermore, a bacterial strain, *Bacillus siamensis* GL-02, isolated from ZDD, was found to significantly affect growth of *F. graminearum*. *In vitro* and *in vivo* assays demonstrated that *B. siamensis* GL-02 had good capability to inhibit *F. graminearum*. These results revealed that *B. siamensis* GL-02 could be a potential biocontrol agent for the control of maize stalk rot.

## Introduction

Maize (*Zea mays*) is an important grain crop for human food security, fodder, and biofuel production. However, its annual yield is significantly decreased due to the plant disease of stalk rot caused by the *Fusarium* spp., which can produce deoxynivalenol (DON) and zearalenone (ZEN) toxins (Quesada-Ocampo et al., 2016; Jedidi et al., 2021; Sumarah, 2022) that are harmful to humans and animals (Trail, 2009; Pestka, 2010; Wang et al., 2020). *F. graminearum* initially grows between living maize cells and later penetrates into the host cells while killing nearby plant cells (Zhang et al., 2016). The initial stage of maize stalk rot occurs in the maize filling stage, and the peak period of the disease ensues from the end of the milk to waxy stage. The roots and stem bases of susceptible maize plants get browned, and irregular brown spots appear between the stem bases. In addition, the stem tissues rot and get browned, soft, and watery, and the stems become empty and loosened. In severe cases, the first and third quarters of the plant begin to rot, and white hyphae or red mildew can be seen in the stalk of the plant. Microscopic observation of the white hyphae can reveal the presence of a large number of oospores of the pathogen. In the late stage of the disease, the ear of the plant droops, and the plant may easily fall and die prematurely. To overcome obnoxious effect of the plant disease, several resistant cultivars and agrochemical pesticides were applied (Shin, 2014). However, producing resistant cultivars is time consuming (Cheng et al., 2019), and the input of the agrochemicals is proved to be harmful to beneficial microbiomes and considerably change the human attitude (Werf, 1996). In this regard, it is obligatory to search for an alternative eco-friendly microbial source to prevent the plant disease.

The inhibitory effect of rhizobacteria plays a role in the growth of plant pathogens. Antagonistic *Bacillus* strains have been frequently isolated and used as biocontrol agents. *B. subtilis* has been reported to inhibit the growth of *F. verticillioides* and accumulation of fumonisin B1 *in vitro* (Cavaglieri et al., 2005). In addition, *F. verticillioides* has been inhibited by spraying *Pseudomonas fluorescens*, thereby controlling maize ear rot and reducing accumulation of fumonisin (Nayaka et al., 2009). *B. siamensis* KCTC 13613T has been found to significantly inhibit the mycelial growth of the plant pathogenic fungi *Rhizoctonia solani* and *Botrytis cinerea* (Jeong et al., 2012). *B. methylotrophicus* TA-1 isolated from rhizosphere has also been reported as a biocontrol agent against maize stalk rot (Cheng et al., 2019). Moreover, *Bacillus*, *Pseudomonas*, *Serratia*, and *Arthrobacter* have been proven to be effective in controlling fungal diseases (Ashwini and Srividya, 2014). These bacteria are able to cleave chitin of the fungal cell wall and degrade mycotoxin for biological control purposes (Mabuchi et al., 2000; Someya et al., 2001; Wen et al., 2002; Huang et al., 2005; Cheng et al., 2019; Xu et al., 2021). In particular, *Bacillus* spp. are also capable of producing spores that are particularly resistant to adverse conditions, and hence, are more beneficial than other microorganisms in the biocontrol of plant pathogens. As the effect of conventional chemical fungicides on controlling stem rot is lower and the ecological problems resulting from their applications are more prominent, prevention and control of stalk rot by biocontrol agents can effectively reduce the impact on environment (Alori and Babalola, 2018).

In the present study, we investigated microbial composition of two rhizosphere soil types by analyzing bacterial 16s rRNA and fungal ITS sequences. To find the inhibitory effect of rhizobacteria, the maize rhizosphere bacteria were separated by dilution coating plate method (Castellano-Hinojosa et al., 2018), and *B. siamensis* was isolated from the rhizosphere soil of the maize affected with severe stalk rot in the field.

## Materials and methods

### Sampling location and samples collection

All rhizosphere soil samples were collected from the field of Dongling county, Shenyang City, Liaoning province, one of the major maize planting areas in China, at the R6 stage of maize. The five-point sampling method was used, and three plants were selected from each sample point. Soil samples were collected from rhizosphere of severe stalk rot diseased plant (ZDD), and rhizosphere soil of the nearest healthy plant from the sampling severe stalk rot diseased plant (ZDH). Litter and soil around the root were removed with a shovel. At a depth of 20–30 cm from the surface, the soil attached to the root was collected and placed in a 50 mL sterilized centrifuge tube. Samples from different sample points were mixed and numbered and brought back to the laboratory in an ice box. All rhizosphere soils were divided into two parts, one part was stored at –80°C for DNA extraction and high-throughput sequencing, and the other was saved in 4°C for bacterial isolation and determination of soil chemical properties.

### Rhizosphere soil chemical properties

The chemical properties of rhizosphere soils were measured, and the effects of soil chemical properties on microbial communities were evaluated using canonical correspondence analysis (CCA). pH was determined by potentiometry. Soil organic matter (SOM) was measured by alkaline hydrolysis diffusion method. Soil available Nitrogen (AN) and available potassium (AK) were measured by Kjeldah method and flame photometry, respectively. Total phosphate (TP) was measured by NaOH-Mo-Sb colorimetry, and available phosphate (AP) were determined by NaHCO3 extraction-Mo-Sb colorimetry.

### DNA extraction and polymerase chain reaction amplification

Total soil DNA were extracted from 0.1 g of soil sample using the E.N.Z.A.™ Soil DNA Kit (Omega, USA) according to the manufacturer’s instructions, and DNA quantity and quality were determined using a NanoDrop 2000 spectrophotometer (Thermo Scientific, USA). DNA was stored at −80°C until further analysis. For each sample, the bacterial 16S rRNA gene was amplified 16S rRNA gene universal primers 338F (5′-ACTCCTACGGGAGGCAGCA-3′) and 806R (5′- GGACTACHVGGGTWTCTAAT-3′) (Lane, 1991), and the ITS region of the fungal rRNA gene was amplified using the fungal-specific primer pair ITS5F (5′- GGAAGTAAAAGTCGTAACAAGG-3′)/ITS2R (5′-GCTGCGTTCTTCATCGATGC-3′). Polymerase chain reaction (PCR) amplification and purification were performed as previously described. The purified PCR products were quantified using a QuantiFluor™-ST system (Promega, USA), and the amplicons were pooled in equimolar ratios for sequencing.

### Illumina MiSeq sequencing and analysis

Amplicon libraries were constructed using the TruSeq Nano DNA LT Library Prep Kit for Illumina (Illumina, USA) following the manufacturer’s recommendations, and index codes were added. The amplicon libraries were sequenced on a MiSeq PE250 sequencer (Illumina, USA), and 300-bp paired-end reads were generated. The resulting paired sequence reads were then merged, trimmed, filtered, aligned, and clustered by operational taxonomic unit (OTU) using USEARCH v. 5.2.236 software. Sequences with ≥ 97% similarity were assigned to the same OTU by the UPARSE-OTU algorithm in QIIME.

Bacterial and fungi community alpha diversity indices, including Good’s coverage, ACE, Chao1, Shannon index and Simpson index were generated using QIIME. The defined OTUs were used to calculate rarefaction curve. For beta diversity, bacterial community composition was analyzed using principal component analysis (PCA). Metabolic functions of bacterial communities were predicted using the PICRUSt (Phylogenetic Investigation of Communities by Reconstruction of Unobserved States) software based on the KEGG database.^1^ Means were compared between samples by the Tukey’s honestly significant difference (HSD) test using IBM SPSS 22. Differences were considered statistically significant at *p* < 0.05.

### Bacterial isolation and screening

After removing the impurities, 5 g of the rhizosphere soil sample from ZDD were weighed and mixed with 45 mL of sterilized water to obtain mother liquor with a concentration of 0.1 g/mL. Following gradient dilution of the mother liquor, 100 μL of the samples were inoculated into nutrient broth (NB) at a concentration of 1 × 10^–7^∼1 × 10^–8^ g/mL and incubated at 28°C. After 24 h, single colonies of different shapes were picked and purified, and the procedure was repeated thrice (Chan et al., 2003). Subsequently, the isolated rhizosphere bacteria were inoculated 3 cm from the center of the potato dextrose agar (PDA) plate containing *F. graminearum* disk. Each treatment was repeated thrice. After being cultured for 6 days at a constant temperature of 28°C, the strain showing the strongest antagonistic effect against *F. graminearum* was named as GL-02 and selected for follow-up study.

The genomic DNA of strain GL-02 was extracted by using bacterial genome extraction kit (Sigma, NA2110). The 16S rRNA gene was amplified by 16S rRNA gene universal primers 27F (5′-AGAGTTTGATCCTGGCTCAG-3′) and 1492R (5′-TACGGYTACCTTGTTACGA-3′) (Lane, 1991). The PCR product was sequenced by the Beijing Genomics Institute, China. The sequences obtained were submitted to the NCBI, and similarity sequences were compared with known sequences in the NCBI database using BLAST (Kim et al., 2012). The evolutionary relationship between the 16S rRNA gene of strain GL-02 and similar species was analyzed by maximum likelihood method using MEGA7.0 software (Kumar et al., 2016), with bootstrap value set to 1,000. Combined with colony morphology, physiological and biochemical indicators (Xue et al., 2006), and alignment results, strain GL-02 was determined to be *B. siamensis*, and was registered and preserved at China General Microbiological Culture Collection Center (CGMCC, No. 16068).

### Antagonism of strain GL-02 against *Fusarium graminearum*

The dual culture assay was used to assess the antagonistic activity of *B. siamensis* GL-02 against *F. graminearum* (Zhou et al., 2011). In simultaneous plating group, an 8-mm-diameter *F. graminearum* disk was placed in the middle of the PDA plate and 10 μL of *B. siamensis* GL-02 culture supernatant were inoculated at two equidistant positions (3 cm) from the fungal disk and incubated at 28°C. Prior to inoculation, *B. siamensis* GL-02 was cultured in Luria–Bertani (LB) medium at 37°C and 220 r/min for 24 h. In staggered plating group, an 8-mm-diameter *F. graminearum* disk was placed in the middle of the PDA plate and incubated at 28°C for 2 days. Then, 10 μL of *B. siamensis* GL-02 strain were inoculated at two equidistant positions (3 cm) from the fungal disk and incubated at 28°C. Prior to inoculation, *B. siamensis* GL-02 was cultured in LB medium at 37°C and 220 r/min for 24 h. The control was prepared by placing an 8-mm-diameter *F. graminearum* disk in the middle of the PDA plate and incubating the plate at 28°C. All the experiments were repeated five times. After incubating the plates at 28°C for 5 days, the diameter of *F. graminearum* colony was observed and measured. The bacteriostatic rate was calculated as follows (Hameeda et al., 2010): [(Control fungal colony diameter-Experimental group fungal colony diameter)/Control fungal colony diameter × 100%].

The biocontrol function gene of *B. siamensis* GL-02 was amplified using specific primers for the five biocontrol function genes (srfAA, bacA, fenD, spaS, ituC, and bmyB) (Mora et al., 2011). The PCR amplification system comprised 25 μL of 1 × EcoTaq PCR SuperMix, 0.4 μM sense and anti-sense primers, and 2 μL of genomic DNA, with a total volume of 50 μL. The PCR conditions were as follows: pre-denaturation at 95°C for 4 min, denaturation at 94°C for 1 min, annealing for 1 min, extension at 70°C for 1 min, and final extension at 70°C for 10 min. The annealing temperature for the genes fenD, ituC, spaS, srfAA, and bacA was 58°C, whereas that for the gene bmyB was 55°C. A total of 20 μL of each amplification product were subjected to electrophoresis (1% agarose gel).

Subsequently, the culture medium and liquid fermentation conditions for *B. siamensis* GL-02 were optimized with respect to inhibition of *F. graminearum* growth. The optimized medium composition was as follows: beef extract, 0.3%; peptone, 1.0%; glucose, 2.25%; magnesium sulfate heptahydrate, 0.01%; and ammonium sulfate, 0.05%. The optimal fermentation conditions were as follows: liquid volume, 50 L (39.6%); inoculum volume, 5%; pH, 8.0; fermentation time, 48 h; rotation speed, 180 r/min; and fermentation temperature, 40°C. The *B. siamensis* GL-02 fermentation broth was mixed with ash (fertilizer) and applied during maize sowing, and maize hybrid Zhengdan 958 seeds were used. The maize seeds were sown in two groups, experimental and control groups. In the experimental group, the *B. siamensis* GL-02 mixture was applied at the time of sowing, whereas the maize grown in the control group were not exposed to the *B. siamensis* GL-02 mixture. The experimental and control groups were strictly separated from each other.

*F. graminearum* spores solution was inoculated into one row of maize at the level of second and third stems before the maize V12 stage. Subsequently, five maize stalks subjected to each treatment were randomly collected at 7, 14, and 21 days to observe *F. graminearum* infection, and the length of the lesion was measured and recorded. The lesion inhibition rate was calculated as follows: [(Control lesion length − Experimental group lesion length)/Control lesion length × 100%].

## Results

### Sequencing results and microbial alpha diversity

In total, Illumina Miseq sequencing generated 1,080,915 quality bacterial 16S rRNA gene sequences and 1,205,999 classifiable fungal ITS gene sequences with range of read length from 133 to 526. The Good’s coverage of each sample, which reflects the captured diversity, was higher than 97.59% for all samples. Rarefaction curves of OTUs at 97% sequence similarity of all samples tended to approach the saturation plateau (Supplementary Figure 1). Therefore, the sequencing depth was adequate for assessing the diversity of bacterial and fungal communities of our samples.

**FIGURE 1:**
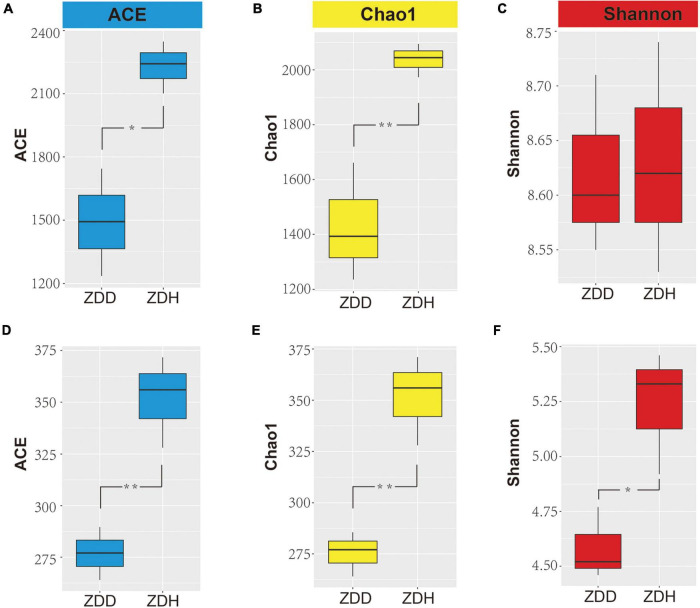
Differences in bacterial **(A–C)** and fungi **(D–F)** alpha diversity indices in maize rhizosphere soil. Panels **(A,D)** were ACE diversity indices, panels **(B,E)** were Chao diversity indices, and panels **(C,F)** were Shannon diversity indices. ZDD refers to rhizosphere soil with severe stalk rot disease, and ZDH refers to the rhizosphere soil with disease-free near by the ZDD.

The alpha diversity indices of bacteria and fungi were represented by the ACE, Chao1, and Shannon indices (Figure 1). The results show the bacterial ACE and Chao1 indices of ZDH were significantly higher than ZDD (*p* < 0.05), while the Shannon index was not significantly different between ZDH and ZDD (*p* > 0.05). Among the fungal communities, ACE, Chao1, and Shannon indices of ZDH were significantly higher than ZDD. In sum, maize stalk rot had significant effects on soil microbial alpha diversity.

### The composition of rhizosphere microbial communities

**FIGURE 2:**
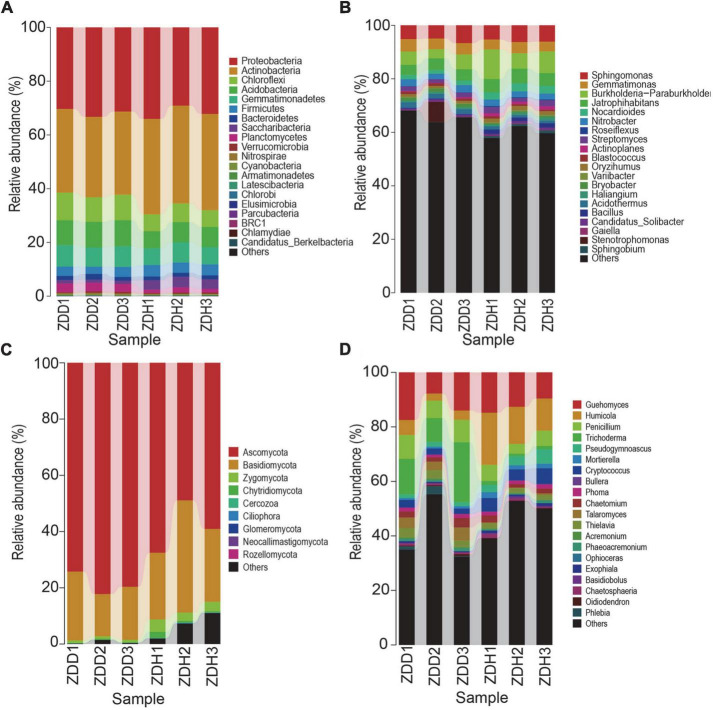
Relative abundances of dominant microbial taxonomic groups in maize rhizosphere soil. **(A)** Dominant bacterial phyla. **(B)** Dominant bacterial genus. **(C)** Dominant fungal phyla. **(D)** Dominant fungal genus. Others represent taxonomic groups with low content or those that are unclassified. ZDD refers to rhizosphere soil with severe stalk rot disease, and ZDH refers to the rhizosphere soil with disease-free near by the ZDD.

Based on the analysis of the top 20 most abundant bacterial phyla, the dominant phyla in samples included *Proteobacteria, Actinobacteria, Chloroflexi, Acidobacteria, Gemmatimonadetes, Firmicutes* (Figure 2A). *Proteobacteria* was the most dominant phylum, accounting for more than 30% of the total number of phyla present, followed by *Actinobacteria* and *Chloroflexi*. In the same habitat, the proportion of each phylum varied between disease soil samples and disease-free soil samples. In the diseased rhizosphere, *Chloroflexi* and *Acidobacteria* accounted for a higher proportion when compared to the disease-free rhizosphere. At the genus level, the dominant bacteria genera of samples included *Sphingomonas, Gemmatimonas, Burkholderia-Paraburkholderia, Jatrophihabitans, Nitrobacter, Roseiflexus and Streptomyces* (Figure 2B). *Sphingomonas, Gemmatimonas, Burkholderia-Paraburkholderia* and *Jatrophihabitans* were the most dominant genera. The top nine most abundant fungi phyla were *Ascomycota, Basidiomycota, Zygomycota, Chytridiomycota, Cercozoa, Ciliophora, Glomeromycota, Neocallimastigomy*, and *Rozellomycota* (Figure 2C). *Ascomycota* was the most dominant phylum. *Guehomyces, Humicola, Penicillium*, and *Trichoderma* were the most dominant fungal genera (Figure 2D).

Venn diagram analysis was conducted to detect exclusive and shared OTUs across samples (Supplementary Figure 2). 1,310 bacterial OTUs were shared in all samples, and there were 887 and 1,386 unique bacterial species which were present in the ZDD and ZDH, respectively. Furthermore, the results showed that the number of fungal OTUs found in the two samples were up to 269. The number of fungal OTUs exclusively found in ZDD and ZDH were 93 and 241, respectively. These indicate that disease decreased the community diversity of bacteria and fungi in rhizospheric soil. Moreover, We can see that the distribution of sequences also demonstrated that each type of soil had its own microbial population.

### Beta diversity of rhizosphere microbial communities

**FIGURE 3:**
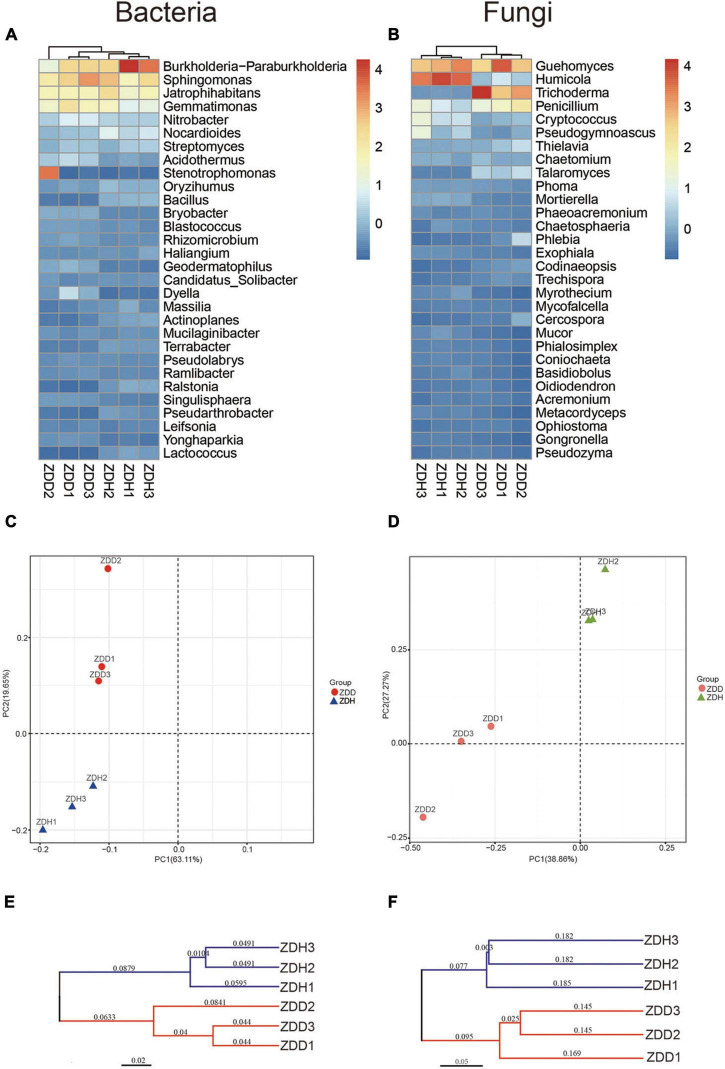
Differences in bacterial **(left panel)** and fungal **(right panel)** community structure of different maize rhizosphere soil samples. Dendrogram and heatmap of soil bacteria **(A)** and fungi **(B)** communities for the top 30 abundant genera. Principal component analyses of soil bacteria **(C)** and fungi **(D)** communities. The cluster tree of soil bacteria **(E)** and fungi **(F)** communities from different rhizosphere soil samples on Bray-Curtis index. ZDD refers to rhizosphere soil with severe stalk rot disease, and ZDH refers to the rhizosphere soil with disease-free near by the ZDD.

The dendrogram and heatmap showed the differences of the top 30 genera from six samples (Figures 3A,B). The relative abundance of bacillus in healthy samples were significantly higher than diseased samples. A principal component analysis (PCA) clearly revealed that the soil microbial community structures varied among samples in both bacterial and fungal community (Figures 3C,D). ZDD and ZDH were clearly separated from each other. Of the total variance in the bacterial dataset, the first two principal components together explained 82.76% of the total bacterial communities. In addition, the first principal component (PC1) was the most important, accounting for 63.11% of the total variation of the bacterial communities. For fungal dataset, 66.13% of the total fungal communities were explained by the first two principal components. A hierarchical clustering tree was constructed to describe and compare the similarities of samples (Figures 3E,F). Based on the similarities between the community compositions, the 6 soil samples were divided into two groups. ZDD and ZDH were separated in the cluster.

**FIGURE 4:**
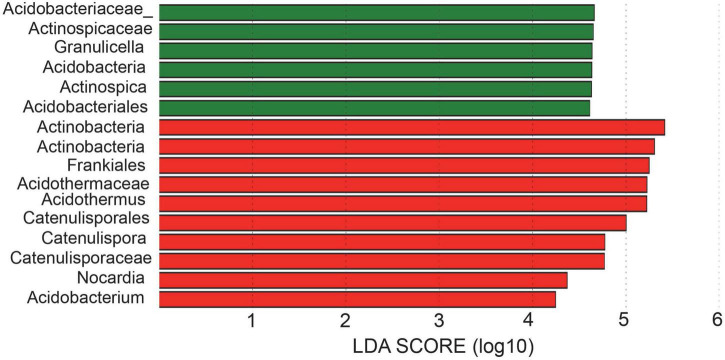
Linear discriminant analysis effect size (LEfSe) for bacterial taxa between soils of healthy and disease.

Bacterial taxa with significantly different abundances were detected by using **LEfSe** among two samples (Figure 4). In total, 2 phyla (*Acidobacteria* and *Actinobacteria*), 2 classes (*Acidobacteria and Actinobacteria*), 4 orders (*Acidobacteriales, Catenulisporales, Corynebacteriales, Frankiales*), 5 families (*Acidobacteriaceae_, Catenulisporaceae, Nocardiaceae, Actinospicaceae, Acidothermaceae*), and 6 genera (*Granulicella, Catenulispora, Nocardia, Acidothermus, Acidobacterium, Actinospica*) showed significant differences among different rhizospheric samples. Specifically, the bacterium and fungus with the highest LDA value in ZDD was Actinobacteria (logarithmic LDA score = 5.77) and Ascomycota (logarithmic LDA score = 5.02), respectively (Supplementary Tables 1, 2). The bacteria with the highest LDA value in ZDH was Acidobacteriaceae_ (logarithmic LDA score = 4.66), and the fungi with the highest LDA value was Chaetomiaceae (logarithmic LDA score = 5.80) (Supplementary Tables 1, 2).

### Function prediction of bacterial communities

**FIGURE 5:**
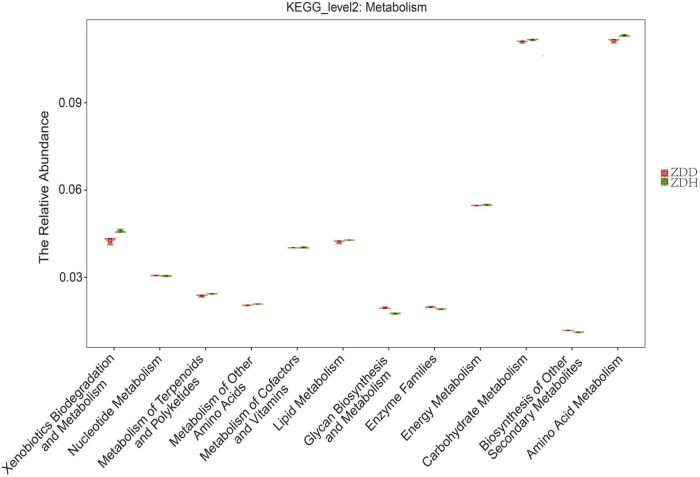
Predicted metabolism functions of the bacterial communities found in maize rhizosphere soil samples. ZDD refers to rhizosphere soil with severe stalk rot disease, and ZDH refers to the rhizosphere soil with disease-free near by the ZDD.

A total of 12 level two KEGG Orthology (KO) groups were identified in the bacterial communities that were involved in metabolism pathways (Figure 5). The result showed that functional gene belonging to carbohydrate metabolism and amino acid metabolism were markedly abundant in the dataset. Among these gene, the relative abundances of genes predicted to be associated with amino acid metabolism, carbohydrate metabolism, and xenobiotics biodegradation and metabolism was significantly higher in disease-free soil than disease soil, while the relative abundances of genes predicted to be associated with Glycan biosynthesis and metabolism was significantly higher in disease soil.

### Correlation between soil physicochemical properties and the bacterial communities

**FIGURE 6:**
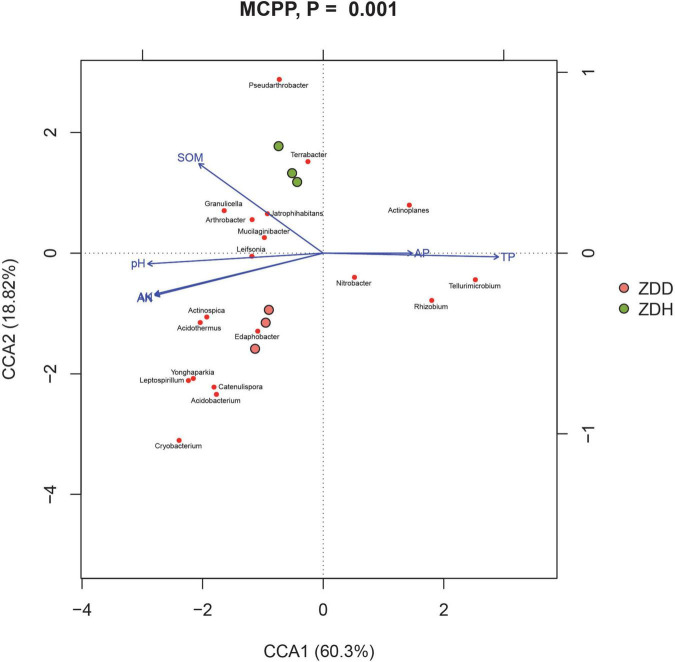
Canonical correspondence analysis (CCA) between the soil and bacterial community in maize rhizosphere soil. ZDD refers to rhizosphere soil with severe stalk rot disease, and ZDH refers to the rhizosphere soil with disease-free near by the ZDD.

The physicochemical properties of soil samples were analyzed by Shanghai Personalbio Technology Co., Ltd., China, and the results were showed in Supplementary Table 3. Canonical correspondence analysis (CCA) was conducted to analyze the association between bacterial community and physicochemical factors (Figure 6). Soil physicochemical factors SOM, PH, available K, and available N positively correlated with each other, while P (available P and total P) negatively correlated with other measured factors. Moreover, these physicochemical factors were significant correlation with bacterial community structure (MCPP, *P* = 0.001).

### Biocontrol function analysis

**FIGURE 7:**
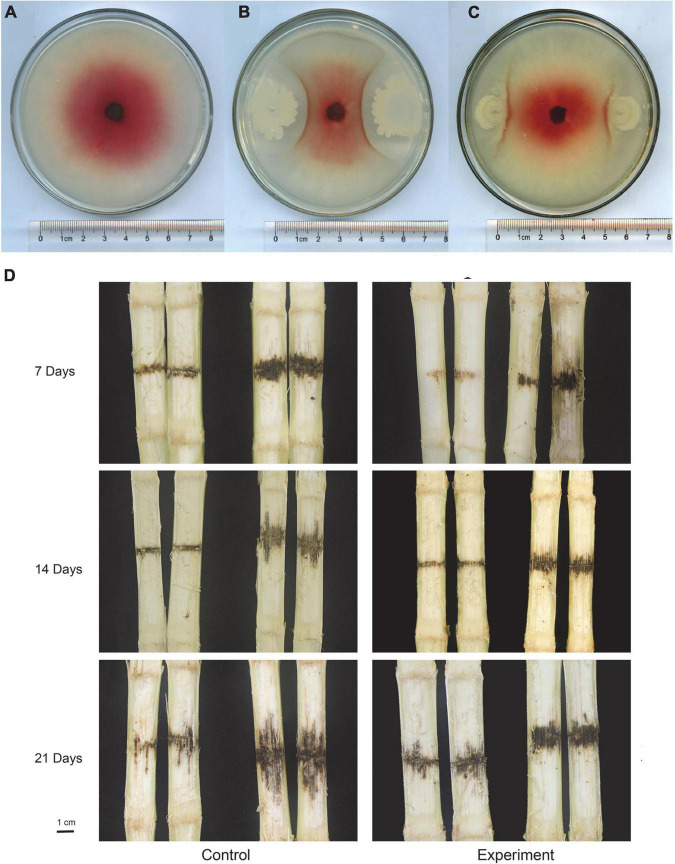
*In vitro* competition assays with *F. graminearum* and *B. siamensis* GL-2 after 5 days of growth. *F. graminearum* control **(A)**, *B. siamensis* GL-02 and *F. graminearum* plated simultaneously **(B)**, *B. siamensis* GL-02 plate 2 days after *F. graminearum* inoculation **(C)**. Pathogenicity of *F. graminearum* after application of a *B. siamensis* GL-2 **(D)**. Left half of panel **(D)** depicts the maize stem that was not inoculated with *F. graminearum*, and the right half indicates the maize stem inoculated with *F. graminearum*.

After incubating the plates at 28°C for 5 days, the diameter of *F. graminearum* colony was observed and measured (Figures 7A–C). The calculated bacteriostatic rate was shown in the Table 1. The rates of inhibition of *F. graminearum* by *B. siamensis* GL-02 in the simultaneous and staggered plating groups were determined to be 77.20 and 43.24%, respectively.

**TABLE 1:** *Fusarium graminearum* growth evaluations showing average, standard deviation, percent inhibition by *B. siamensis* GL-02, and *p*-value (unpaired *t*-test).

	Lesion length 1 (cm)	Lesion length 2 (cm)	Lesion length 3 (cm)	Lesion length 4 (cm)	Lesion length 5 (cm)	Average (cm)	Standard deviation	Inhibition%	*P*-value
Dual culture (simultaneous plating)	1.648	2.044	2.494	1.53	1.608	1.865	0.361399	77.20%	4.06E-09
Dual culture (staggered plating)	4.65	4.752	4.706	4.58	4.52	4.642	0.083651	43.24%	1.60E-08
Control	8.17	8.362	8.484	7.61	8.268	8.179	0.302746	–	–

The concentration of conidia was quantified using a hemocytometer and diluted to 1 × 10^6^ spores/ml for inoculation. Maize stem was inoculated at the second or third internode using a sterile micropipet tip above the soil line, followed by injection of 20 μl macroconidia suspension. *F. graminearum* spores solution was inoculated into one row of maize at the level of second and third stems before the maize V12 stage. Subsequently, five maize stalks subjected to each treatment were randomly collected at 7, 14, and 21 days to observe *F. graminearum* infection, and the length of the lesion was measured and recorded (Figure 7D). The lesion inhibition rate was calculated as follows: [(Control lesion length − Experimental group lesion length)/Control lesion length × 100%] (Table 2). The results obtained showed that the lesion inhibition rate was 5.35, 43.75, and 37.21% at 7, 14, and 21 days, respectively.

**TABLE 2:** Lesion length evaluations showing average, standard deviation, percent inhibition by *B. siamensis* GL-02, and *p*-value (unpaired *t*-test).

	Group	Lesion length 1 (cm)	Lesion length 2 (cm)	Lesion length 3 (cm)	Lesion length 4 (cm)	Lesion length 5 (cm)	Lesion length 6 (cm)	Average (cm)	Standard deviation	Inhibition%	*P*-value
7 Days	Experiment	1.6	2	2.2	2.5	3	2.5	2.3	0.4396969	5.35%	0.6714278
	Control	3.5	2	2.1	2	2.5	2.5	2.43	0.5217492	–	
14 Days	Experiment	2	2.6	2.2	3.5	2.1	2.2	2.43	0.5120764	43.75%	2.02E-05
	Control	4.5	4.1	4.2	4.6	4.5	4	4.32	0.2266912	–	
21 Days	Experiment	3.1	3.9	4.5	4.3	4	4.5	4.05	0.4821825	37.21%	0.0002794
	Control	5.6	6.4	5.5	8.1	6.4	6.7	6.45	0.8578073	–	

## Discussion

The pathogen-infected host plants would attract beneficial microbes to protect themselves (Gao et al., 2021). This so-called “cry for help” strategy of host plants was proved across in different species (Bakker et al., 2018; Liu et al., 2019, 2020; Gao et al., 2021). For example, based on the evidence of the wheat rhizosphere, an infection with a soil-borne pathogen *Fusarium pseudograminearum* has driven the recruitment of beneficial microbes *Stenotrophomonas rhizophila* to boost host plant defense (Liu et al., 2021). Therefore, we selected severe diseased rhizosphere soil but not healthy rhizosphere soil to isolate biocontrol agents.

Plants have to endure abiotic and biotic stresses since they cannot move (Zhang et al., 2022). Rhizosphere microbial interactions are complex and are important to plant health and growth (Liu et al., 2019). Soil microorganisms play critical roles in soil fertility, plant productivity, and plant immunity (Saleem et al., 2019; Pascale et al., 2020). In particular, rhizosphere microorganisms are essential for uptake of nutrients, suppression of pathogen colonization, and maintain root-associated microecological balance (Bakker et al., 2018; Liang et al., 2019), and it is increasingly recognized that some rhizosphere microbiomes can offer new opportunities to be used as biological control factors to control plant disease (Del Barrio-Duque et al., 2019; Li et al., 2020). The analysis of microbial diversity in the rhizosphere used high-throughput sequencing has been effective in pilot studies (Liang et al., 2019; Veach et al., 2019; Ju et al., 2020; Zhou et al., 2020). In this study, we characterized the rhizosphere microbial community composition across disease and disease-free soil samples. The bacterial ACE and Chao1 indices of disease-free soil were significantly higher than disease soil (*p* < 0.05). All three fungal alpha diversity indices of health soil samples were significantly higher than disease samples (Figure 1). *Proteobacteria*, *Actinobacteria*, *Chloroflexi*, *Acidobacteria*, *Gemmatimonadetes*, *Firmicutes* comprised the largest components of each rhizosphere community. *Proteobacteria* were the dominant phylum of rhizosphere community, which is consistent with researches of rhizosphere communities of other crops. For example, Proteobacteria were the primary bacterial taxa observed in the rhizosphere of *Saccharum officenarum L.* (Gao et al., 2019), *Cucumis sativus L.* (Jin et al., 2019), *Triticum aestivum* (Jochum et al., 2019), which suggested that *Proteobacteria* was the most common phylum in soils globally because they generally grow fast like weedy species and are well known to respond to unstable carbon sources (Liang et al., 2019). Among *Bacillus* spp., *B. subtilis*, *B. laterosporus*, *B. cereus*, *B. licheniformis*, *B. thuringiensis*, *Paenibacillus polymyxa*, *B. pumilus*, etc., have been mainly employed for plant disease control (Leelasuphakul et al., 2008; Swain and Ray, 2009).

It is well known that information on the functional analysis is important for understanding the role of bacterial diversity (Huang et al., 2015; Lee et al., 2019; Liang et al., 2019). The relative abundances of genes predicted to be associated with amino acid metabolism, carbohydrate metabolism, and xenobiotics biodegradation and metabolism was significantly higher in disease-free soil than disease soil. Our results highlighted that functional trait on metabolism pathway were different between disease and disease-free rhizosphere soil samples. The disease maize selected microbes with specific functional genes related with “Glycan biosynthesis and metabolism.”

*B. siamensis* GL-02 was isolated from corn rhizosphere. In our study, the maize rhizosphere bacteria were separated by dilution coating plate method. *B. siamensis* GL-02 was isolated and confirmed to be effective in inhibiting the growth of *F. graminearum*. It has been demonstrated to have significant prospective application in many fields (Yang et al., 2015). What’s more, it also exhibits strong *in vitro* fungi toxicity as well as induces defense response in plants to reduce plant disease severity (Gu et al., 2017). Our research has shown that GL-02 can directly inhibit the growth of *F. graminearum* and can be potentially used as a biocontral agent in plant protection. It is well known that *Bacillus* spp. produce a wide array of antagonistic compounds, which can direct inhibit pathogens (Fira et al., 2018). Previous studies indicated that *Bacillus* spp. could produce lipopeptide antibiotics to inhibit pathogen infection (Zhu et al., 2019). According to chemical structure, iturins, fengycins, and surfactins are the three major families of lipopeptides (Bonmatin et al., 2003; Ongena and Jacques, 2008). These lipopeptides can inhibit phytopathogen growth and also can stimulate host defense (Ongena and Jacques, 2008). The next step will be try to highlight the biological control mechanism of GL-02 and find the active compounds involved.

## Data availability statement

The data presented in this study are deposited in the National Center for Biotechnology Information databases, accession number: PRJNA739021.

## Author contributions

All authors performed conceptualization, methodology, writing—original draft preparation. All authors contributed to the article and approved the submitted version.

## Footnotes

1
http://www.genome.jp/kegg/pathway.html

## Supplementary material

Supplementary Figure 1 Bacterial **(A)** and fungi **(B)** rarefaction curves for all samples at a 97% OTU sequence similarity threshold.

Supplementary Figure 2 Venn diagram of unique and shared operational taxonomic units (OTUs) among samples. Numbers indicate the number of unique and shared bacterial **(A)** and fungal **(B)** OTUs.

Supplementary Figure 3 Linear discriminant analysis effect size (LEfSe) for fungal taxa between soils of healthy and disease.

Supplementary Table 1 Diseased and health soil differential bacteria.

Supplementary Table 2 Diseased and health soil differential fungi.

Supplementary Table 3 Results of soil physicochemical properties.
