# Supplementary figures and images for: Maize stalk rot caused by Fusarium graminearum alters soil microbial composition and is directly inhibited by Bacillus siamensis isolated from rhizosphere soil
Source: Front Microbiol. 2022 Oct 20;13:986401. doi: 10.3389/fmicb.2022.986401 (PMC9630747; doi:10.3389/fmicb.2022.986401)

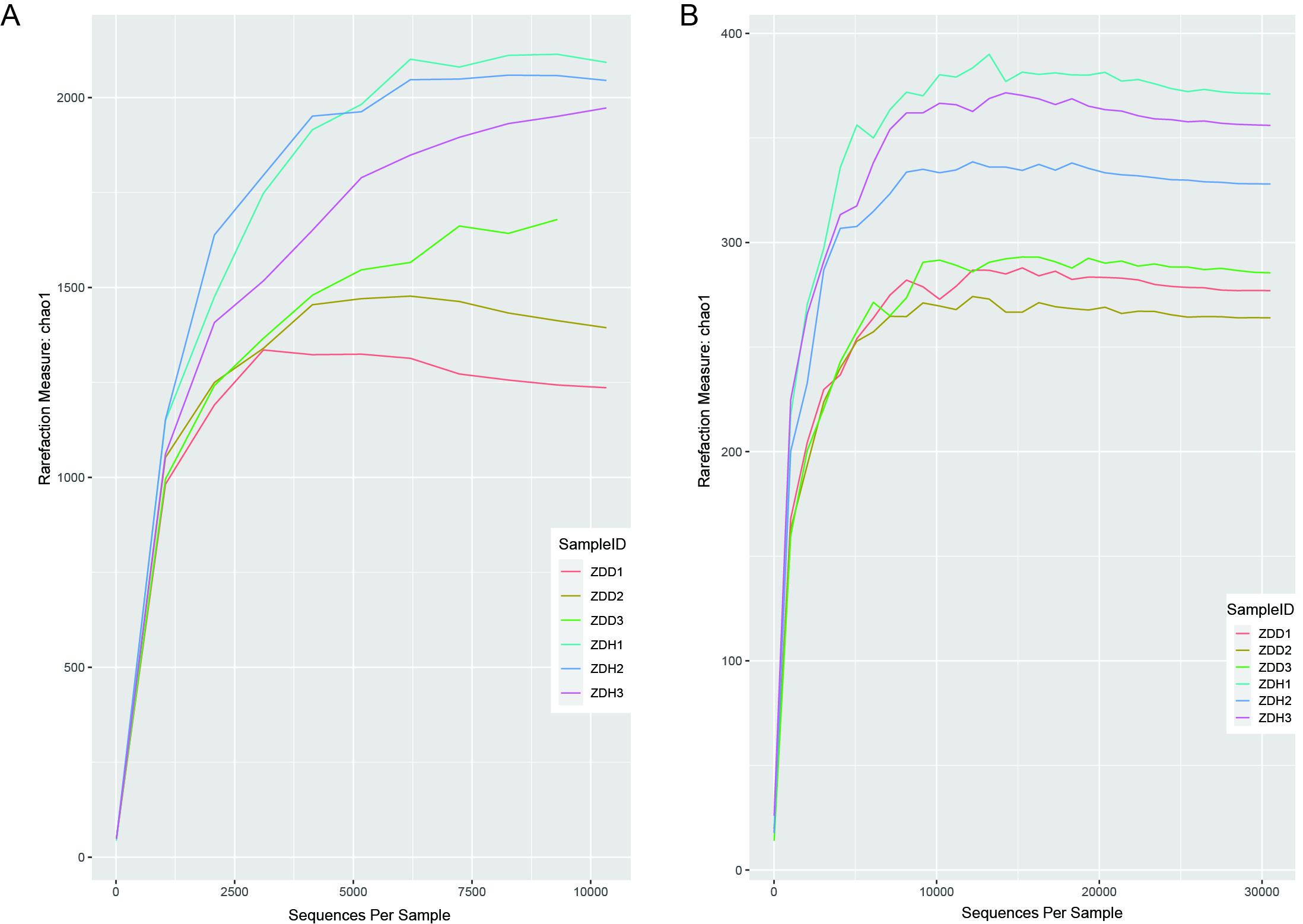

Supplement: Supplementary Figure 1 — Bacterial (A) and fungi (B) rarefaction curves for all samples at a 97% OTU sequence similarity threshold. [file Image_1.JPEG]

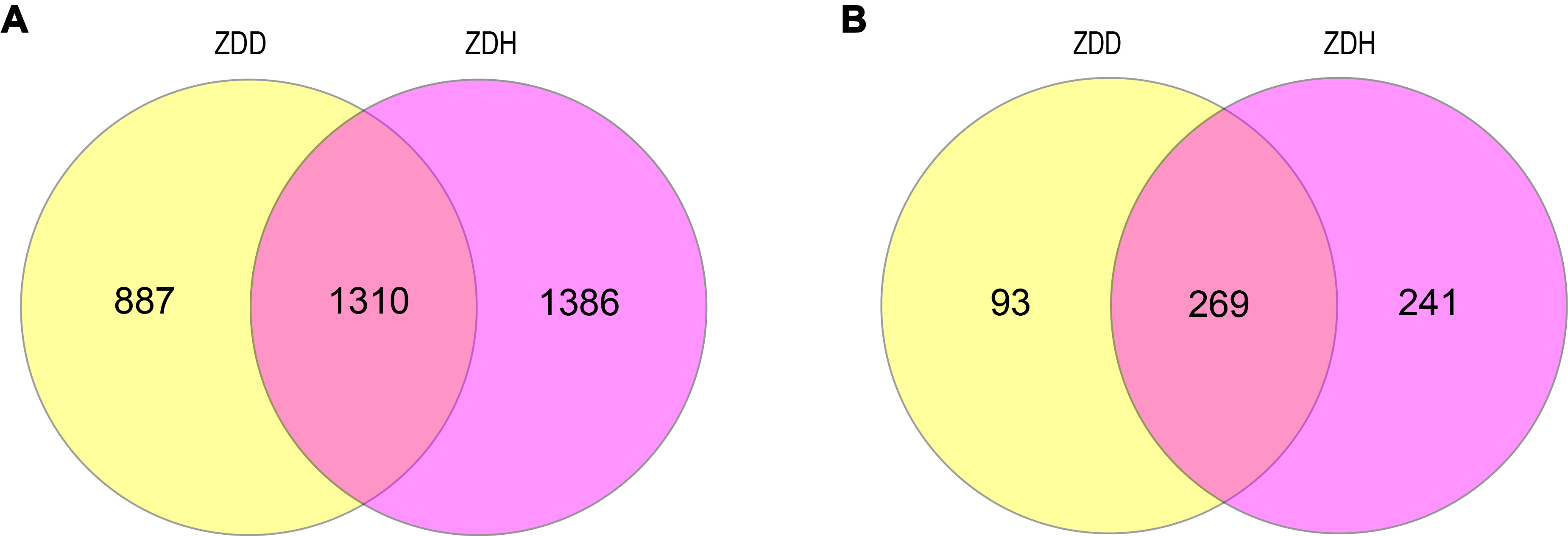

Supplement: Supplementary Figure 2 — Venn diagram of unique and shared operational taxonomic units (OTUs) among samples. Numbers indicate the number of unique and shared bacterial (A) and fungal (B) OTUs. [file Image_2.JPEG]

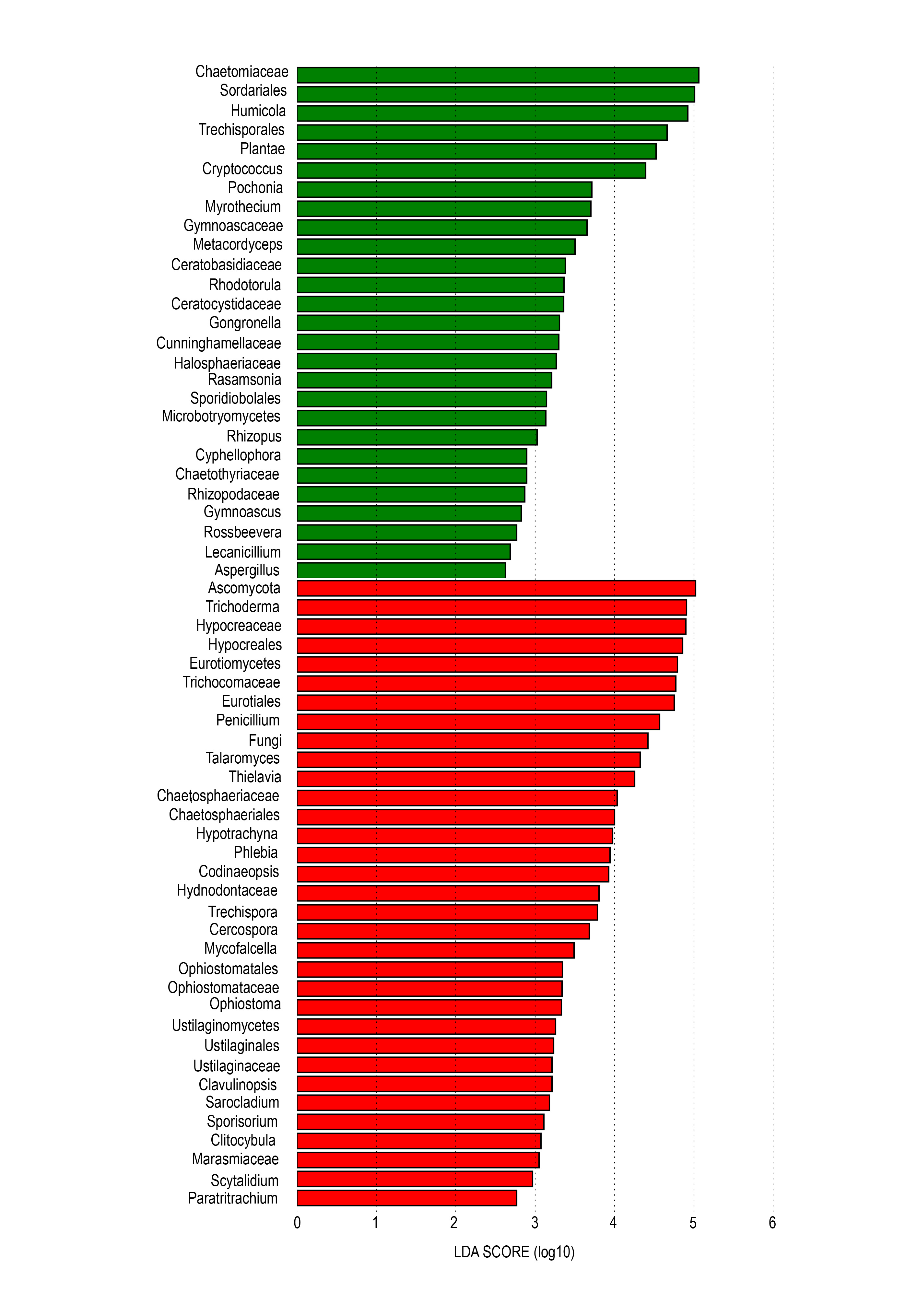

Supplement: Supplementary Figure 3 — Linear discriminant analysis effect size (LEfSe) for fungal taxa between soils of healthy and disease. [file Image_3.JPEG]
